# Effect of Milling on the Mechanical Properties of Chopped SiC Fiber-Reinforced ZrB_2_

**DOI:** 10.3390/ma6051980

**Published:** 2013-05-15

**Authors:** L. Pienti, D. Sciti, L. Silvestroni, S. Guicciardi

**Affiliations:** CNR-ISTEC, National Research Council of Italy-Institute of Science and Technology for Ceramics, Via Granarolo 64, I-48018 Faenza, Italy; E-Mails: laura.pienti@libero.it (L.P.); laura.silvestroni@istec.cnr.it (L.S.), stefano.guicciardi@istec.cnr.it (S.G.)

**Keywords:** milling, chopped fiber, fracture toughness, strength

## Abstract

This work aims at studying the effect of the milling conditions on the microstructure and mechanical properties of a ZrB_2_-5 vol% Si_3_N_4_ matrix reinforced with chopped Hi-Nicalon SiC fibers. Several composites were obtained using different milling conditions in terms of time, speed and type of milling media. The composites were prepared from commercial powders, ball milled, dried and shaped, and hot pressed at 1720 °C. Their relative bulk densities achieved values as high as 99%. For each material the fiber length distribution, the extent of reacted fiber area and matrix mean grain size were evaluated in order to ascertain the effects of milling time, milling speed and type of milling media. While the fracture toughness and hardness were statistically the same independently of the milling conditions, the flexural strength changed. From the results obtained, the best milling conditions for optimized mechanical properties were determined.

## 1. Introduction

Ultra-high temperature ceramics (UHTCs) based on the ZrB_2_-system are of particular interest because of their combination of refractory and engineering properties such as high melting point, relatively low density, high hardness, high stiffness and high strength. These properties make them suitable materials for high temperature applications in refractory industries and especially in aerospace field. 

In the last years, research has focused on the fabrication of dense ZrB_2_ composites possessing high strength, further increased by the use of SiC or MoSi_2_, as typical sintering aids (500–1000 MPa) [1,2,3,4,5]. In particular, the addition of SiC is well known to improve the fracture strength, due to grain refinement, and the oxidation resistance, due to the formation of a protective silica layer [1,2,6]. Despite this positive aspect, the low fracture toughness of these composites, around 2–4 MPa·m^1/2^, limits the spectrum of potential applications. In that regard, it has been reported that a method for increasing their fracture toughness is the addition of discontinuous elongated reinforcement such as SiC whiskers [7,8,9,10,11], or SiC fibers [8,12] that gives promising results in order to solve this problem: fracture toughness values of 6.6–8.5 MPa·m^1/2^ had been obtained. 

The control of the critical parameters in the production of the ZrB_2_ composites, such as dimension and purity of the starting phases, the mixing procedure and the sintering conditions, has a major role in order to avoid degeneration of the reinforcement [9], their agglomeration [12] or breaking [13]. A good dispersion of chopped fibers into the matrix allows the achievement of a homogeneous microstructure and consequently good mechanical properties; proper sintering conditions permit to avoid or limit the degeneration of the reinforcing elements. Recent works have however shown that the addition of chopped SiC fibers increases the fracture toughness, but causes a decrease of strength [9]. Moreover, the relationships between strength and toughness in these composites are not well understood. In previous works [14], it was shown that composites containing 20 vol% of SiC fibers have strength values ranging from 400 to 680 MPa, the most striking difference being the dimensions of the fibers in the sintered materials. Other works [15] have shown that samples with identical composition have significantly different values of flexural strength, but again no definitive explanation for this behavior was found. 

In this work, ZrB_2_-5 vol% Si_3_N_4_ materials reinforced with 15 vol% of SiC chopped fibers were produced using different milling conditions. In particular, the mixtures were produced using different milling time, milling speed and type of milling media. The aim was to study how the milling process affects the distribution of fiber length and how the distribution of fiber length in turn affects the microstructure and the mechanical properties. 

## 2. Experimental Procedure

The composites were produced starting from commercial powders:

- ZrB_2_ Grade B (H.C. Stark, Germany), specific surface area 1.0 m^2^/g, impurity max content: C 0.25 wt%, O 2 wt%, Fe 0.1 wt%, Hf 0.2 wt%, particle size range 0.1–8 µm;
- α-Si_3_N_4_ Baysind (Bayer, Germany), specific surface area 12.2 m^2^/g, impurity max content: O 1.5 wt%;
- SiC chopped fibers (Hi-Nicalon) diameter 14 µm, length 1 mm, 1–5 wt% vinyl alcohol polymer with vinyl acetate, wt% Si:C:O = 62:37:0.5.

The composition of the material consisted in a matrix of ZrB_2_ + 5 vol% Si_3_N_4_, where silicon nitride was added to promote the densification, mixed with 15 vol% of SiC chopped fibers. The powder mixtures were dispersed in absolute ethanol inside a polyethylene bottle and ball milled using different milling conditions:

- milling time: 12, 24 and 48 h;
- milling speed: 130, 200 and 300 rpm;
- milling media: ZrO_2_ or SiC.

A fixed powder/milling media/solvent weight amounts (1:1:1) was adopted. The sizes of ZrO_2_ milling media were 15, 10, 5 mm, and the weight ratio among the three dimensions was kept constant. The available sizes of SiC media were 14 and 7 mm. Several samples were produced in order to study the influence of specific milling parameters. The combination of milling conditions is reported in Table 1. The comparison among samples labeled as A, B and C allows evaluating the influence of milling time; comparison among samples B, D and E the influence of milling speed and comparison between samples B and F the influence of milling media, being SiC much harder than ZrO_2_. 

After milling, the slurries were dried in a rotary evaporator and the powders were shaped in four centimeters diameter pellets. The pellets were debonded at 500 °C for 1 h and hot pressed at 1720 °C, with a holding time of 10 min, in low vacuum (100 Pa) using an induction-heated graphite die with an uniaxial pressure of 40 MPa during the heating and increased up to 50 MPa at the maximum sintering temperature; free cooling followed. After sintering, the bulk densities were measured by Archimedes’ method. 

**Table 1:** List of the samples: label, composition, milling time, milling speed, milling media type, sintering conditions (T*_MAX_*, dwell time, pressure) and densities (theoretical, experimental, relative).

Label	Composition (vol%)	Milling time (h)	Milling speed (rpm)	Milling media	Sintering (°C, min, MPa)	Th. density (g/cm^3^)	Exp. density (g/cm^3^)	Rel. density (%)
ZB [9]	ZrB_2_ + 5 Si_3_N_4_	-	-	-	1700,15,30–50	5.95	5.89	99.0
A	ZrB_2_ + 5 Si_3_N_4_ + 15 SiCf	12	130	ZrO_2_	1720,10,40–50	5.45	5.18	94.9
B		24	130	ZrO_2_	1720,10,40–50	5.45	5.34	97.9
C		48	130	ZrO_2_	1720,10,40–50	5.45	5.40	99.0
D		24	200	ZrO_2_	1720,10,40–50	5.45	5.37	98.4
E		24	300	ZrO_2_	1720,10,40–50	5.45	5.28	96.9
F		24	130	SiC	1720,10,40–50	5.45	5.31	97.3

The microstructure of the samples was analyzed using scanning electron microscope (SEM, Cambridge S360, Cambridge, UK) and energy-dispersive spectroscopy (EDS, X-Act, INCA Energy 300, Oxford Instruments, Abingdon, UK) on fracture and polished surfaces. 

Sections of the sintered materials were cut and polished with diamond paste to 0.25 µm perpendicularly to the hot pressing direction, due to the fact that the fibers tend to align their long axis on this plane. This criteria, which certainly underestimates the actual fiber length, was adopted just for comparative purposes. The fiber length and mean ZrB_2_ grain size were determined on micrographs of polished sections using image analysis (Image-Pro Analyzer 7.0, Media Cybernetics, Silver Spring, MD, Rockville, USA). Length data were collected by image analysis considering only objects with dimensions greater than 10 µm. This limit was assumed in order to avoid data gathering relative to SiC debris, which were present in the sintered microstructure. The mean grain size of ZrB_2_ grains was calculated by the circle method on the polished sections. Using commercial software (MATHEMATICA version 8, Wolfram Inc., Chicago, IL, USA), the grain size data were fitted with the cumulative distribution function (CDF) of a lognormal distribution. Once ordered in a crescent way, a cumulative probability P(Xi) = (i − 0.5)/N, where N is the total number of data [16], was assigned to each value Xi of grain size. The CDF curve of the lognormal distribution was then fitted to this set of points {Xi,P(Xi)} for each material. Fitting a CDF avoids the problem of interval size when one works with frequency values trying to fit a probability distribution function (PDF) [17]. At least one hundred grain size data were taken. For the fiber length, after creating the data sets {Xi,P(Xi)} as described above, a 3-parameter CDF Weibull distribution [18] with a cut-off value of 10 µm, see explanation above, was fitted the each data set. At least 1000 values were collected for each sample. For visual purposes, however, the fitted CDF distributions will be displayed in the following in their PDF form. 

From the original billet, specimens 25 × 2 × 2.5 mm^3^ were cut and machined according to the European Standard prEN 843-1 “Advanced Technical Ceramics – Monolithic Ceramics – Mechanical Properties at Room Temperature – Part 1: Determination of Flexural Strength”. The long axis of the bars and their thickness were kept perpendicular to the hot pressing direction. This orientation of the specimens was thought to give the highest value of strength as the short fibers, which could act as critical flaws, are aligned in the less harmful way. The fracture toughness K*_Ic_* was evaluated using chevron-notched beams (CNB) in flexure. The test bars 25 × 2 × 2.5 mm^3^ (length by width by thickness, respectively) were notched with a 0.1 mm-thick diamond saw. In addition, in this case the long axis of the bars and their thickness were kept perpendicular to the hot pressing direction. The chevron-notched tip depth and average size length were about 0.12 and 0.80 of the bar thickness, respectively. The specimens were fractured using a semi-articulated silicon carbide in four-point fixture with a lower span of 20 mm and an upper span of 10 mm using screw-driven load frame (Instron mod. 6025, Instron, Illinois Tool Works Inc., Norwood, MA, USA). The specimens were loaded with a crosshead speed of 0.05 mm/min. The “slice-model” equation of Munz *et al.* [19] was used to calculate K*_Ic_*. On the same machine and with the same fixture, the flexural strength at room temperature σ*_RT_* was measured on test bars 25 × 2 × 2.5 mm^3^ (length by thickness by width, respectively). Comparing the fracture mode of the specimens for the fracture toughness and the strength it can be seen that the fracture does not propagate on the same plane for the two types of specimen. However, since the short fibers should be isotropically distributed on the plane perpendicular to the hot pressing direction plane, no direction effect should be observed for these two different directions of fracture propagation. The experimental analysis of the effective interaction between the fracture propagation plane and the fiber orientation is demanded to a future work. Finally, the Vickers micro-hardness HV1.0 was measured with a load of 9.81 N using a standard micro-hardness tester (Zwick 3212, Zwick, Ulm, Germany). Ten indentations were randomly placed onto the perpendicular/parallel media polished surfaces for each material. The interaction of the crack front with the microstructure was analyzed by introducing cracks onto polished surfaces with a 98.1 N indentation. 

## 3. Results and Discussion

### 3.1. Densification

All sintering cycles were conducted in the same conditions. No significant difference was noticed in the shrinkage behavior (not shown). The relative densities were expressed as the ratio of the experimental and the theoretical densities. The theoretical densities were calculated with the rule of mixtures, considering the nominal starting compositions and taking 6.10 g/cm^3^, 3.19 g/cm^3^ and 2.74 g/cm^3^ as the density of ZrB_2_, Si_3_N_4_ and SiC fibers, respectively. As reported in Table 1, the relative densities were higher than 96% and reached the value of about 99% for sample C. It is worth noticing that these values can be even underestimated because of the presence of glassy phases derived by the sintering aid reaction to form a Si-O-N amorphous phase. 

### 3.2. Microstructure

**Figure 1:**
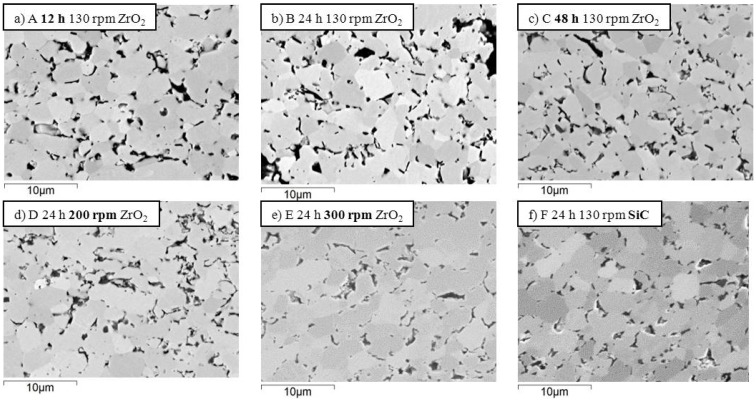
SEM-images of the polished sections of samples: ZrB_2_ matrix. The dark features correspond to secondary phases (amorphous Si-O-N, ZrN and BN).

In the SEM images of Figure 1, the polished sections of the samples A-F are displayed. In these pictures, just the ZrB_2_ matrix is shown. All the materials were nearly fully dense. Dark features correspond to secondary phases formed during sintering, *i.e.*, amorphous Si-O-N, ZrN and BN. The values of mean grain size and relative dispersion are reported in Table 2. As for the mean grain size of ZrB_2_ matrix, it can be noticed that samples A-F had a similar value. There is a slight tendency of the mean grain size to increase with the spread of the fiber length distribution. It was also observed that samples milled with ZrO_2_, A-E, generally have a more faceted shape than those milled with the SiC media, F, suggesting a possible rounding off of the ZrB_2_ powder due to the higher hardness of silicon carbide. 

**Figure 2:**
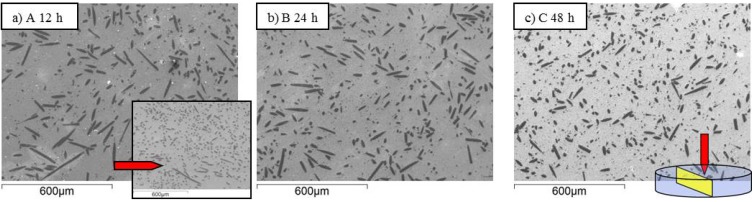
(**a**)–(**c**) SEM-images of samples milled for different times showing the section perpendicular to the hot pressing direction, see labels. The inset in (a) shows the fiber distribution in a section parallel to the hot pressing direction.

**Figure 3:**
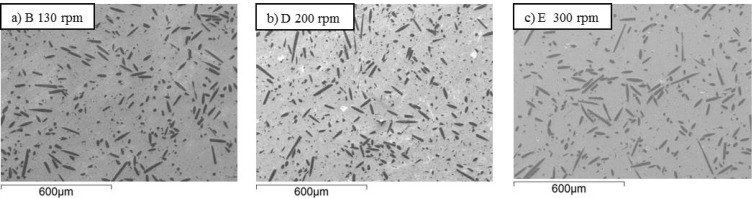
(**a**)–(**c**) SEM-images of samples milled at different speeds, see labels. The polished sections were cut perpendicularly to the hot pressing direction.

**Figure 4:**
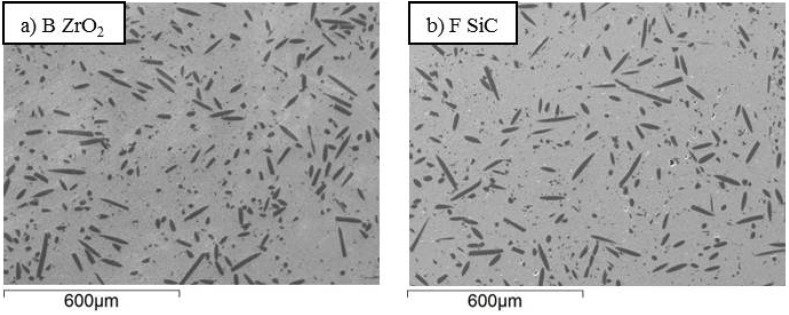
(**a**), (**b**) SEM-images of samples milled with different milling media, see labels. The polished sections were cut perpendicularly to the hot pressing direction.

Fiber dispersion into the matrix is shown in Figure 2, Figure 3 and Figure 4 for samples A-F. Generally speaking, no agglomeration was observed with an orientation distribution of the fibers almost isotropic in the section plane oriented perpendicularly to the hot pressing direction. This can be compared with the anisotropic distribution of fibers shown in the inset of Figure 2a when the section was cut parallel to the hot pressing direction. As expected, the fibers showed the tendency to align their long axis perpendicular to the direction of applied pressure. In Figure 2, Figure 3 and Figure 4, the effects of milling time, milling speed and milling media type on the fiber length can be directly evaluated. At a first glance, it is apparent that a gradual decrease of the fiber length with the increase of milling time occurred (compare sample A, B and C in Figure 2). As for the milling time, the increase of milling speed up to 200 rpm effectively reduced the fiber size, but a further increase to 300 rpm did not lead to any additional improvement (compare sample B, D and E in Figure 3). Also for the milling media, the difference among sample B and F are not so evident only observing the SEM-images (compare sample B and F in Figure 4). 

**Figure 5:**
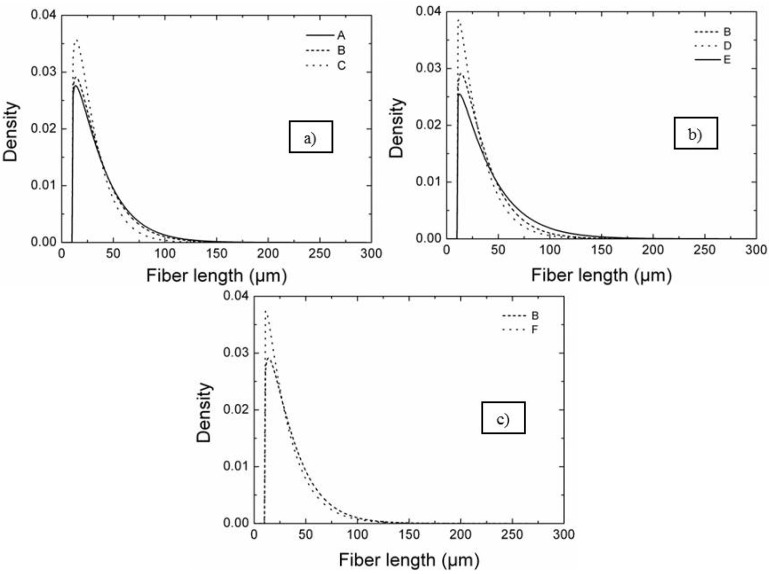
Probability density function curves: (**a**) effect of milling time on the length distribution (sample A, B and C); (**b**) effect of milling velocity (sample B, D and E); (**c**) effect of milling media hardness (sample B and F).

The calculated parameters of the fitted CDF curves are listed in Table 2. Besides the mean grain size, the maximum length, the form factor m the scale factor s_0_, also the main quantiles are reported. The parameters m and s_0_ are indicative of the data dispersion (the higher is m the lower is the data dispersion) and the data median. The fitted CDF curves are shown in Figure 5 as PDF curves grouped according to the different milling parameters. In Figure 5a, the effect of milling time on the length distribution can be evaluated for samples A, B and C. As can be seen, the length distribution became narrower increasing the milling time, as indicated by the increasing of the form factor m from A, milled for the shortest time, to C, milled for the longest time (Table 2). At the same time, the median values of fiber length decreased with increasing milling time passing from 29 µm of sample A to 21 µm of sample C, as indicated by the scale factor s_0_. As a consequence, the density of long fibers decreased with the increase of the milling time, as concluded from the SEM images (sample A, B and C in Figure 2).

**Table 2:** Sample label, mean grain size (m.g.s.), maximum fiber length (l*_max_*), form factor (m), scale factor (s_0_) and quantiles.

Label	m.g.s. ^1^ (µm)	l*_max_* (µm)	Form factor ^2^ m	Scale factor ^2^ s_0_ (µm)	Quantiles (µm)		
					¼	½	¾
ZB [9]	-	-	-	-	-	-	-
A	2.9 ± 1.4	296	1.106 ± 0.004	29.07 ± 0.07	19.42	30.87	49.01
B	2.6 ± 2.0	248	1.126 ± 0.004	27.03 ± 0.05	18.93	29.51	46.12
C	2.3 ± 1.5	239	1.157 ± 0.004	21.50 ± 0.04	17.33	25.67	38.51
D	2.3 ± 1.4	233	1.068 ± 0.003	21.72 ± 0.04	16.76	25.41	39.49
E	2.3 ± 1.1	256	1.065 ± 0.002	33.13 ± 0.04	20.28	33.48	55.03
F	2.2 ± 1.9	274	1.054 ± 0.003	22.93 ± 0.05	17.03	26.19	41.26

^1^ mean ± 1 standard deviation; ^2^ value ± 1 standard error.

In Figure 5b, the fiber length PDF curves of samples B, D and E were compared to study the effect of milling speed. Initially, the distribution width decreased passing from sample B, milled at 130 rpm, to sample D, milled at 200 rpm. Further increasing the milling speed had an opposite effect. The median value of fiber length of sample E milled at 300 rpm increased and the long fiber density increased. Very likely, increasing too much the rotation speed decreased the frictional interaction between the bottle wall and milling media, thus reducing the milling effect as the milling media dropped from a lower height. 

The milling media hardness effect can be evaluated by comparing the PDF curves of samples B and F (Figure 5c), in which the powders mixtures were milled with ZrO_2_ milling media (B) and with SiC milling media (F). As can be seen, increasing the milling media hardness decreased the median fiber length, which passed from 27 µm to 22 µm for sample B and F, respectively, and also the fiber length dispersion. 

Besides the main parameters of the distributions, the maximum fiber length was measured for all the composites (Table 2) and it can be observed that the values were related to the severity of milling procedure. For instance the specimen milled in the mildest conditions, 12 h at 130 rpm (A), resulted in the longest fiber length, about 300 μm. This value showed the tendency to decrease for increasing milling time, from 300 to 240 μm (samples A, B, C). As for the other conditions, the maximum length decreased when increasing the speed from 130 to 200 rpm, but then increased for further increase of the milling velocity. The use of ZrO_2_ instead of SiC milling media resulted in maximum fiber length of 250 and 275, respectively, which is quite surprisingly owing to the different hardness, but could be explained with a highest range of dimensions down to smaller size of ZrO_2_ media, 15, 10, 5 mm *vs.* 14, 7 mm for SiC. No significant correlation was observed between l*_max_* and any of the parameter distributions (m, s_0_, and quantiles). 

**Figure 6:**
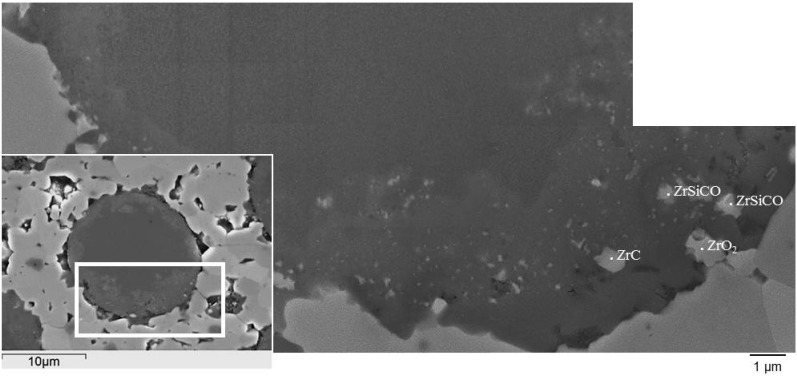
Multilayered core-shell morphology of a SiC fiber in a ZrB_2_ matrix.

Another microstructural feature analyzed was the interface between matrix and fiber. During densification, a strong interface form between the matrix and SiC fibers due to ZrB_2_/SiC fiber interaction [9]. In the sintered materials, the fibers show multilayered core-shell morphology (Figure 6):

- the inner part was constituted of β-SiC crystallites, amorphous Si-C, and turbostratic carbon;
- the surrounding shell was coarsened β-SiC crystallites with embedded ZrO_2_, ZrC, ZrSi_2_, Zr-C-O phases;
- the outermost jagged layer was made of SiC platelets.

This arrangement is due to complex chemical reactions occurring during sintering related to the fiber chemical instability at temperatures higher than 1500 °C. Fiber evolution during sintering is presently under analysis as the extent of modification may be affected by several factors, including sintering temperature/holding time/type of sintering aid. 

The chemical composition and extension of the core/rim structure was analyzed and no variation of the transformed area was found for the different milling conditions. In all cases, the area ratio between the unreacted SiC core and the transformed rim was approximately 70:30. 

### 3.3. Mechanical Properties

The mechanical properties of the ZrB_2_-based composites are shown in Table 3, together with the unreinforced baseline material [9]. 

**Table 3:** Mechanical properties: fracture toughness (K*_Ic_*), flexural strength (σ*_RT_*), Vickers microhardness (H*_V1.0_*). Mean ± 1 standard deviation.

Label	K*_Ic_* (MPa·m^1/2^)	σ*_RT_* (MPa)	H*_V1.0_* (GPa)
ZB [9]	3.8 ± 0.1	600 ± 90	13.4 ± 0.6
A	5.5 ± 0.4	355 ± 12	11.8 ± 1.3
B	5.4 ± 0.4	389 ± 18	13.0 ± 0.8
C	5.2 ± 0.1	389 ± 24	13.0 ± 0.8
D	5.1 ± 0.2	415 ± 12	12.1 ± 0.9
E	5.6 ± 0.4	369 ± 13	13.9 ± 0.8
F	5.11 ± 0.02	368 ± 27	13.5 ± 1.0

The Vickers hardness of these composites ranges between 11.8 and 13.9 GPa. It should be however considered that the indentation marks at 9.81 N were not large enough to test both matrix and fibers so that the measured values mainly represent the hardness of the matrix. No clear relationships between hardness and milling conditions were identified. More than the milling conditions, matrix density and matrix grain size can have influenced the hardness results. 

**Figure 7:**
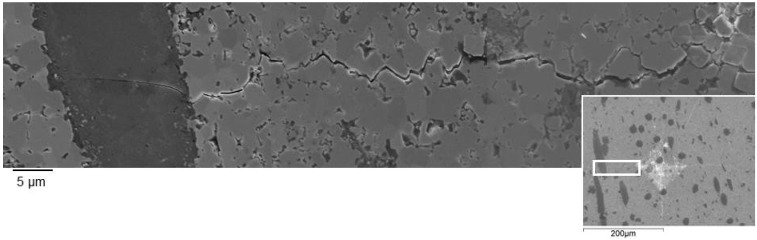
Crack path generated by a 98.1 N indentation.

The fracture toughness of ZrB_2_-based composites (5.1–5.6 MPa·m^1/2^) did not significantly change for the samples milled in different milling conditions, but increased of about 45% in comparison with the unreinforced materials (3.8 MPa·m^1/2^ [9]). The Analysis of Variance (ANOVA) performed on the toughness data confirmed that there was no significant difference among samples A–F. Figure 7 shows an example of crack path generated by a 98.1 N indentation. The crack crossed the fibers without any significant deflection, as a consequence of the strong matrix/fiber interface, which did not allow fiber debonding. Crack pinning and residual stresses were thus considered the dominant toughening mechanisms for this type of composites. However, due to the unfavorable mismatch between the thermal expansion coefficients of the matrix and the fibers [20,21], the residual stresses give in this case a negative contribution according to the model of Taya *et al.* [21]. Therefore, crack pinning must be considered the major toughening mechanism in these composites. From our experimental results, there was no influence of the length distribution of the SiC fiber on the fracture toughness. 

**Figure 8:**
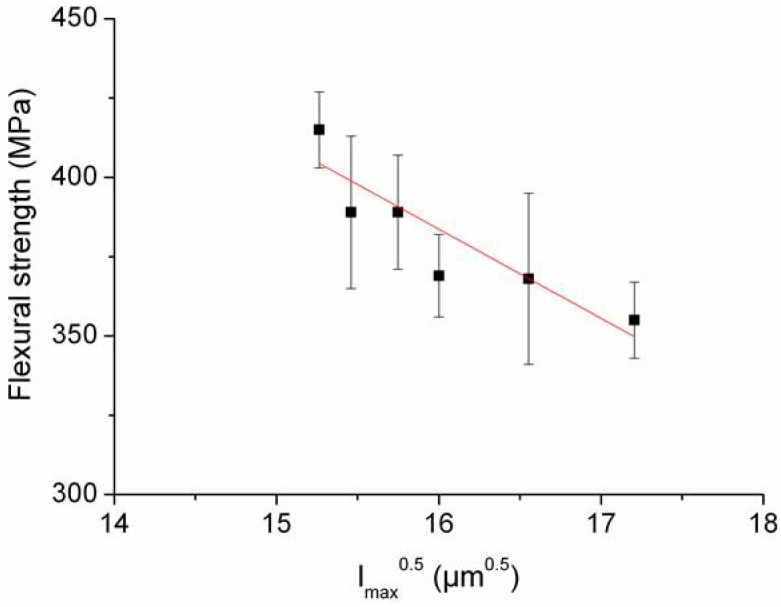
Strength *vs.* square root of maximum fiber length, considering the longest fiber as a critical flaw.

On the contrary, the ANOVA performed on the strength data (ranging from 355 to 415 MPa) indicated a significant difference among samples A–F. However, very loose correlations were found between strength and the fitted parameters of the length distributions. The best correlation was observed when the strength was evaluated against the square root of maximum fiber length (see Figure 8), considering the longest fiber as a critical flaw. 

**Figure 9:**
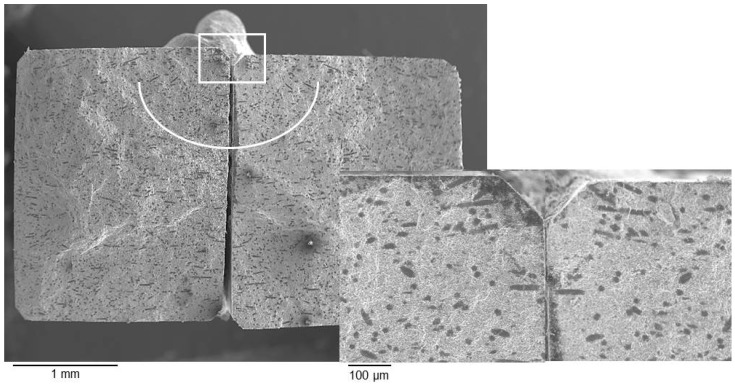
Fracture surface of sample D where the short fibers are aligned perpendicular to the tensile surface.

However, when observing the fracture surfaces of the samples, we did not find any fiber, which was at the origin of the sample failure. Moreover, as can be seen on the fracture surfaces, an example of which are shown in Figure 9, the short fibers are aligned perpendicular to the tensile surface. According to the Newman and Raju [22], this is an unfavorable orientation for the fiber to act as critical flaw. With the simplifying hypothesis that the fracture origins were located on the tensile surface of the specimens with a half-penny shape of radius *c*, the size *c* of the critical flaws can be estimated according to Equation (1) [23]:

(1) $$\sigma =\sqrt{\frac{2}{\pi }}\frac{K_{Ic}}{\sqrt{c}}$$

where *σ* is the fracture strength and *K_Ic_* the fracture toughness. Considering the values of Table 3, the calculated size *c* of the critical flaw for samples A–F were 153, 123, 114, 96, 147 and 122 µm, respectively (for comparison, the starting matrix has a critical flaw size *c* of 26 µm). No feature with dimensions matching these values was observed on the fracture surfaces of the composites. It is therefore apparent that the fracture in this type of composites is somehow related to the introduction of the fibers, as the composite strength dropped in spite of the toughness increase with respect to the starting matrix, and that the critical flaws are not the fibers *per se* but must be the result of the fiber/matrix and fiber/fiber interaction. An example of this could be regions where fibers tend to aggregate. In Figure 8, such regions are outlined and it is possible to see that the distance between neighbor fibers in these regions is much shorter than the average. Since the fracture toughness is the same, the strength of the composites seems to be therefore determined by the size of these fiber aggregates, which form during processing. However, as the residual stress in the fibers is compressive, very likely it is the matrix residual stress, which can explain the strength variation in our composites. According to a micromechanical model [24], the residual stress in the matrix just outside a short fiber is a function of three factors: (1) elastic and thermal expansion mismatch between fiber and matrix, (2) fiber volume and (3) L/d, where L and d are the long and transverse dimension of the fiber, respectively. In composites with elastic and thermal properties of the fibers and matrix as those we are considering, the radial component of the residual stress in the matrix just outside the fiber is compressive while the tangential component is tensile. The maximum value of the latter is attained just at the ends of the fiber. Of particular relevance for our composites is factor (3) since factor (1) and (2) do not vary among the composites. As shown in Reference [24], the higher is the aspect ratio (L/d) of the fiber, the higher is the value of the matrix tangential tensile stress at the fiber ends. This can explain the apparent relationship that was observed between the flexural strength and the fiber maximum length, Figure 8. Moreover, the possible superposition of the residual thermal stress field around neighbor fibers can also explain why fractography indicates fibers aggregates as fracture origins: high values of residual tensile stress in the matrix can be locally generated by the superposition of the tangential tensile stress around agglomerated fibers. The flexural strength is therefore determined by the combined effect of the external applied tensile stress and this local residual tensile stress. 

## 4. Conclusions

SiC chopped fiber-reinforced ZrB_2_ ceramics were produced using different milling conditions, in terms of milling time, milling speed and type of milling media, with the aim of studying how the milling process affects the distribution of fiber length and how the distribution of fiber length in turn affects the microstructure and the mechanical properties. 

Different milling conditions for the production of these ceramics affect the distribution of the fiber length and therefore the microstructure and the mechanical strength of the composites. 

From the image analysis on polished surfaces, it was observed that the average length of the fibers decreases with the increasing of milling time and hardness of milling media, from 300 to 240 µm (samples A–C) and from 275 to 250 µm (samples B and F), respectively. As for milling speed, our results seem to indicate that speed cannot be increased too much in order to avoid a counter effect on the milling efficacy. The matrix grain size seems slightly coarser for the composite processed in the milder milling conditions. The fibers interact with the ZrB_2_ matrix evolving to a multilayered core-shell morphology constituted of a SiC core and a transformed crown in which ZrC and Zr-Si-C-O phases were found. According to evidences, it was ascertained that different milling conditions did not affect the extent of fiber transformation. 

The fracture toughness and hardness of the composites were statistically the same, covering toughness values from 5.1 to 5.5 MPa·m^1/2^ and hardness values up to 13.9 GPa, independently of the milling conditions. On the other hand, the flexural strength showed differences, which were not related to any parameter of the fiber length distribution, but to the maximum fiber length. The fracture origins are supposed to be fiber aggregates. 

From our results, the best milling conditions were 24 h, 130/200 rpm with ZrO_2_ or SiC as milling media, *i.e.*, materials B, D and F. 
